# 

**Published:** 1900-05-05

**Authors:** 


					j n ju akvmi iiax/*
i 11c"oMIcitfjfPS"or"V*TgiiUaL pm*?j
CADBURY'S ?f> If f* CADBURYS
COGOA m> - " ^ 1 NO DRUGS OR ALKALIES USED.
ABSOLUTELY PORK,
No. 710.JVol. XXVIII. [jftggSaturday,
May 5, 1900.
rSubscnption Terms, 1 p^JCE TWOPENCE.
800 p? oU. ?J

				

